# High efficiency *Agrobacterium*‐mediated site‐specific gene integration in maize utilizing the FLP‐*FRT* recombination system

**DOI:** 10.1111/pbi.13089

**Published:** 2019-03-28

**Authors:** Ajith Anand, Emily Wu, Zhi Li, Sue TeRonde, Maren Arling, Brian Lenderts, Jasdeep S. Mutti, William Gordon‐Kamm, Todd J. Jones, Nicholas Doane Chilcoat

**Affiliations:** ^1^ Agricultural Division of Dow DuPont Corteva Agriscience™ Johnston IA USA

**Keywords:** *Agrobacterium*, co‐integrate vector, FLP/*FRT*, maize transformation, site‐specific integration, RMCE

## Abstract

An efficient *Agrobacterium*‐mediated site‐specific integration (SSI) technology using the flipase/flipase recognition target (FLP/*FRT*) system in elite maize inbred lines is described. The system allows precise integration of a single copy of a donor DNA flanked by heterologous *FRT* sites into a predefined recombinant target line (RTL) containing the corresponding heterologous *FRT* sites. A promoter‐trap system consisting of a pre‐integrated promoter followed by an *FRT* site enables efficient selection of events. The efficiency of this system is dependent on several factors including *Agrobacterium tumefaciens* strain, expression of morphogenic genes *Babyboom* (*Bbm*) and *Wuschel2* (*Wus2*) and choice of heterologous *FRT* pairs. Of the *Agrobacterium* strains tested, strain AGL1 resulted in higher transformation frequency than strain LBA4404 THY‐ (0.27% vs. 0.05%; per cent of infected embryos producing events). The addition of morphogenic genes increased transformation frequency (2.65% in AGL1; 0.65% in LBA4404 THY‐). Following further optimization, including the choice of *FRT* pairs, a method was developed that achieved 19%–22.5% transformation frequency. Importantly, >50% of T0 transformants contain the desired full‐length site‐specific insertion. The frequencies reported here establish a new benchmark for generating targeted quality events compatible with commercial product development.

## Introduction

Advancements in plant transformation technology have made it possible to insert DNA sequences into plant genomes with relative ease. Methods have been developed for transformation of a large number of plant species, most often using *Agrobacterium*‐mediated or biolistic methods (De Buck *et al*., 2009; Jackson *et al*., 2013; Kohli *et al*., 2003; Wu *et al*., 2014; Zhi *et al*., 2015). However, transgenic events obtained from a single DNA construct, referred to as sister events, often exhibit different phenotypes (Hobbs *et al*., 1990; Jones *et al*., 1987; Peach and Velten, 1991). This variation led to a transgenic trait development paradigm wherein large numbers of sister events are generated and subjected to phenotyping, in order to identify an event with the desired phenotype (Strauss and Sax, 2016).

Most transformation methods generate sister events with significant differences at the DNA level, including copy number, truncation and rearrangement (Afolabi *et al*., 2004; Maessen, 1997; Puchta *et al*., 1994). This accounts for some event‐to‐event phenotypic variation. In order to minimize event‐to‐event variation, typically ~70% of the T0 events produced from the simple constructs (1–2 transgenes) are discarded using the molecular criteria for quality events (Zhi *et al*., 2015) and even higher proportions of the events are discarded when complex constructs are used (Anand *et al*., 2018). Therefore, only quality events (QE; single copy vector backbone‐free events) are preferred for phenotyping.

Additionally, deregulation of transgenic events that disrupt an endogenous gene is difficult (EFSA GMO Panel, 2011). Therefore, it is a common practice to discard events with transgenes in or near endogenous genes. Since 60%–75% of otherwise quality events fail to meet the above criteria, cumulatively >90% of the transgenic T0 events are discarded (Anand and Jones, 2018). One possible approach to reduce this attrition is to accurately insert transgenes into well characterized insertion sites through site‐directed integration (Akbudak *et al*., 2010; Cardi and Neal Stewart, 2016; Rinaldo and Ayliffe, 2015).

Most commercial transgenic maize products contain multiple transgenes. These multiple traits are combined through trait introgression and this process is increasingly difficult and costly with more traits (Peng *et al*., 2014; Sun and Mumm, 2015). A transformation method that uses site‐directed integration to create transgenic events in a single genetic region, a so‐called complex trait locus (CTL), presents a possible solution (Chilcoat *et al*., 2017). In a CTL, multiple transgenic events, each inserted in a short genomic region (1–5 centimorgans), can be linked through crossing. Once linked, these multiple traits segregate together, and can be treated as a single locus when conducting trait introgression.

The preferred methods for site‐directed insertion have typically relied on site‐specific integration (SSI) or homologous recombination (HR) (Lyznik *et al*., 2003; Ow, 2007; Terada *et al*., 2002; Tzfira and White, 2005). HR has only been achieved at very low frequencies (Srivastava and Thomson, 2016). SSI may have the potential for higher efficiencies since it relies on recombinases to insert donor DNA. The most preferred SSI strategy employs a target genomic locus that contains two heterologous recombination target sites (RT) flanking a selectable marker gene and donor DNA (Albert *et al*., 1995; Schlake and Bode, 1994; Turan *et al*., 2010, 2013). In the presence of a recombinase, the donor DNA is exchanged with the target, a process referred to as recombinase‐mediated cassette exchange (RMCE). This approach has been applied to transgene insertion in plants (Ebinuma *et al*., 2015; Li *et al*., 2009; Louwerse *et al*., 2007; Nandy and Srivastava, 2011, 2012; Nanto and Ebinuma, 2008; Nanto *et al*., 2005, 2009; Srivastava and Thomson, 2016).

Site‐specific integration in plants has generally been achieved using biolistic delivery of both the donor DNA and DNA encoding the recombinase, usually in the form of circular plasmid DNA (Albert *et al*., 1995; Chawla *et al*., 2006; Srivastava and Ow, 2002). However, biolistic delivery of DNA often results in complex DNA integration (multiple copies of the transgenes, truncated DNA and large deletion of genomic DNA). Most T0 events do not contain the desired RMCE. In short, even though SSI has been reported in a number of studies, low SSI frequencies (Li *et al*., 2009; Nanto *et al*., 2005; Ow, 2002), combined with the complex nature of DNA integrations, have prevented this technology from being widely used.

Here, we describe an efficient method of SSI in elite maize inbreds. This method uses *Agrobacterium*‐delivery of the flipase/flipase recognition target (FLP/*FRT*) recombinase system and the maize *Babyboom* (*Bbm*) and maize *Wuschel2* (*Wus2*) genes (Lowe *et al*., 2016) to improve plant regeneration. The effect of *Agrobacterium* strain, vector design, use of *Bbm*/*Wus2* genes and choice of heterologous *FRT* pairs on transformation frequency and recovery of T0 RMCE events in two elite maize inbreds is described. The method described here is a step‐function improvement over previously described maize transformation methods for generating quality events that do not disrupt endogenous genes.

## Results

### Development and characterization of recombinant target lines

**Figure 1:**
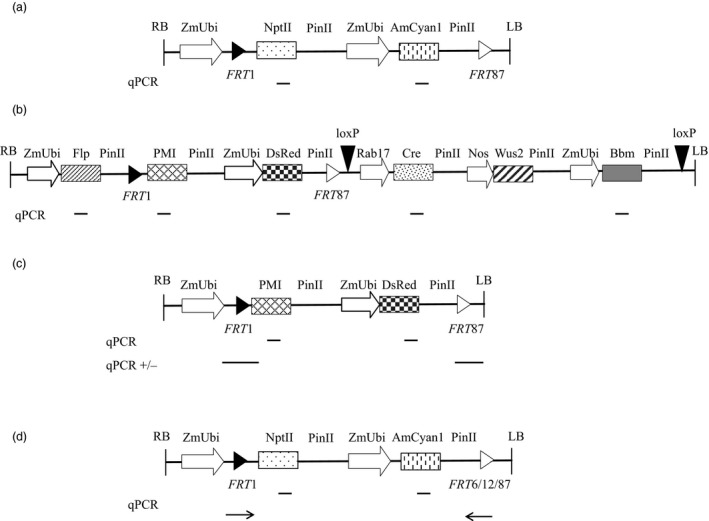
Schematic presentation of the DNA constructs and the intended recombinase‐mediated cassette exchange event (RMCE). (a) Target T‐DNA containing the constitutive promoter ZmUbi driving the neomycin transferase (*nptII*) gene as plant selectable marker, and the same ZmUbi promoter driving the cyan fluorescent (*AmCyan1*) gene as fluorescent marker for selecting transformed cells. A *FRT*1 site (black triangle) is placed between the ZmUbi promoter and the nptII gene, and a *FRT*87 site (white triangle) is placed at the 3′ end. (b) Donor DNA3 T‐DNA containing the same heterologous *FRT* sites flanking a promoterless phosphomannose isomerase (*pmi*) gene, which confers mannose resistance when expressed and a fluorescent reporter gene, *DsRed*, driven by ZmUbi promoter allowing the selection of recombined transgenic events is shown as an example of a donor construct. This donor construct also contains the ZmUbi promoter driving the *flp* gene delivering the FLP recombinase needed for generating intended RMCE events on the 5′ of the donor DNA, an inducible *cre* gene by Rab17 promoter, a maize Wuschel (*Wus2*) gene driven by a nos promoter and a maize Babyboom (*Bbm*) gene driven by ZmUbi promoter on the 3′ end of the donor DNA flanked by *loxP* sites (inverted black triangles). Transient expression of the *flp*,* Wus2* and *Bbm* gene is sufficient for recovering RMCE events. (c) RMCE event is essentially the target DNA, wherein the *nptII* and *AmCyan1* gene between the *FRT*1 and *FRT*87 site is replaced with the *pmi* and *DsRed* gene on the donor DNA. The *pmi* gene is activated upon being inserted downstream of the ZmUbi promoter following cassette exchange between the *FRT* sites. All the components outside the *FRT* sites on the donor DNA are not integrated following recombination in an intended RMCE event. (d) The qPCR assay devised to quantify cross‐reactivity between different heterologous *FRT* sites. Relative positions of the gene‐specific qPCR assays, genomic DNA border‐specific PCR assays are marked with straight lines which were used for quantifying corresponding expression units and *FRT* junction calls, while the line with arrow indicate the relative position of the primer‐probe used for detecting excision.

Recombinant target lines (RTL) were created with heterologous *FRT* pairs consisting of a ZmUbi promoter followed by a *FRT*1 site; neomycin phosphotransferase (NPTII) selectable marker; cyan fluorescent protein (AmCyan1) marker; and a second, heterologous, *FRT* site (*FRT*6, 12 or 87; Figures 1a and 1d). A representative construct with *FRT*1*/*87 is described in Table S1. The ZmUbi promoter serves as a promoter trap when the RTL is subsequently used for RMCE. This enables activation of a marker gene in the donor DNA only when RMCE has occurred. This design is similar to one described for soybean SSI (Li *et al*., 2009, 2010). The RTLs were molecularly characterized using Southern‐by‐Sequencing™ technology, hereafter referred to as SbS analysis (Zastrow‐Hayes *et al*., 2015). SbS utilizes capture‐based target enrichment of samples prior to next‐generation sequencing (NGS) and is used to determine the insertion sequence and intactness of the inserted DNA at the target site. SbS allows the selection of single copy backbone‐free events with known genetic insertion sites. In addition, this technique can also confirm the absence of unintended DNA sequence insertion, either at the target site or other genomic locations.

### SSI optimization in target line GT6

Once RTLs were created, we conducted a series of experiments with immature embryos derived from hemizygous plants to optimize SSI efficiency. The donor T‐ DNA design included a promoter‐less selectable marker gene, *pmi*, and red fluorescent protein marker gene *DsRed* flanked by corresponding heterologous *FRT* sites that matched the target FRT sites and a *flp* expression cassette (Donor DNA1, Table S1). In some cases, the donor T‐DNA also carried morphogenic genes, and an inducible Cre cassette as described in Figure 1b. Upon delivery of the donor DNA and expression of the FLP recombinase, RMCE can occur, wherein the RTL containing *nptII* + *AmCyan1* is replaced with *pmi* + *DsRed* (Figure 1c) carried on the donor DNA. As a result, the promoter‐less *pmi* gene is inserted downstream of the ZmUbi promoter allowing retransformed events to be selected on mannose containing medium.

**Figure 2:**
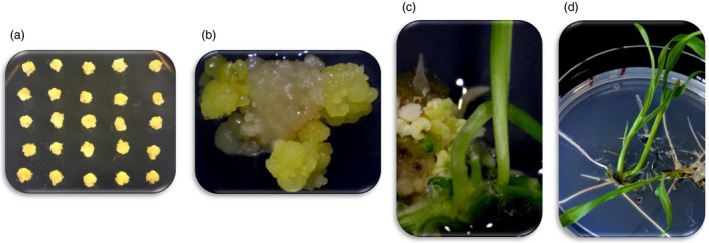
The different stages in transformation for selecting intended RMCE events using the target line GT6. (a) Retransformation of immature embryos from the RTL containing the *nptII* selectable marker. (b) Selection of the putative RMCE events in media supplemented with mannose; this selection requires 2–3 rounds of transfer before a site‐specific integration event is identified. (c) Regeneration of the putative SSI event after three rounds of selection in mannose supplemented media and, (d) Rooting of the putative RMCE events in media supplemented with mannose. The overall transformation process to generate putative RMCE events takes over 3 months.

We evaluated two different *Agrobacterium* strains (LBA4404 and AGL1) and five different T‐DNA vector designs (Table S1) for optimizing SSI frequency. Initial studies for selecting *Agrobacterium* strain, optimizing construct design and optimizing culture conditions involved a single RTL in the elite genotype HC69 with *FRT*1*/*87 *FRT* sites (GT6). The process included *Agrobacterium* infection of immature embryos (Figure 2a), two or three rounds of mannose selection (Figure 2b), regeneration of transgenic events (Figure 2c) and rooting of the individual transgenic event on selection media (Figure 2d).

Once T0 events were regenerated, we identified events with the intended RMCE outcome through a combination of quantitative PCR (qPCR) and PCR analyses. This included a multiplexed PCR assay for detection of the vector backbone. PCR assays were also designed to sequences flanking the *FRT* sites and qPCR assays targeted to the excised marker gene (*nptII*), genes from the donor DNA (e.g. *pmi* and *DsRed*) and the FLP recombinase gene (*flp*) (Figure 1 and Table S2). RMCE events were defined as having the (i) presence of single intact copies of the donor genes (*pmi* and *DsRed*); (ii) absence of the excised marker gene (*nptII*); (iii) presence of *FRT*1 and *FRT*87 junctions; and (iv) absence of unintended DNA sequence insertions including the vector backbone and the *flp* gene. Some events have cassette exchange but also may contain additional DNA insertions, for example random insertions of the T‐DNA or backbone sequences. Other events may have accurate recombination of the 5′ *FRT* site but illegitimate recombination at the 3′*FRT* site. These events are considered non‐RMCE events.

### *Agrobacterium* strain and morphogenic genes

It was previously shown different *Agrobacterium* strains vary in their ability to transform maize (Cho *et al*., 2014; Li *et al*., 2015). Therefore, we evaluated two commonly used *Agrobacterium* strains, AGL1 (a succinamopoine‐type hypervirulent strain) and LBA4404 THY‐ (an auxotrophic octopine‐type strain) for SSI. AGL1 and LBA4404 THY‐ carrying the donor DNA 1 (Table S1), were used. The T0 transformation frequency is presented in Table 1 indicating that the strain AGL1 resulted in greater than fivefold higher transformation frequency than strain LBA4404 THY‐.

**Table 1:** Effect of *Agrobacterium* strain and maize morphogenic genes *Bbm* and *Wus2* on transformation frequency and RMCE event recovery in maize inbred HC69 (GT6) with *FRT*1/87 target site

Maize inbred	*Agrobacterium* strain	*Bbm/Wus2*	Embryos (number)	Events (number)		T0 frequency (percentage)	
				T0	SSI	Transformation	RMCE*
HC69	AGL1	−	3376	9	4	0.27	0.12
	AGL1	+	3436	91	38	2.65	1.12
	LBA4404 THY‐	−	4015	2	0	0.05	0
	LBA4404 THY‐	+	3953	24	5	0.61	0.13

* RMCE events are characterized by (i) presence of single intact copy of the donor genes (*pmi* and *DsRed*); (ii) absence of the excised marker gene (*nptII*); (iii) presence of *FRT*1 and *FRT*87 junctions; and (iv) absence of unintended DNA sequence insertions including those derived from vector backbone, *Bbm*,* cre* and *flp* gene.

The molecular event quality was determined as previously described. Molecular analysis of the T0 events and the frequency of SSI events recovered from the two strains from multiple experiments are presented in Table 1 and Table S2. Molecular analysis of the T0 events indicated the two T0 events regenerated from LBA4404 THY‐ were non‐RMCE events, while four out of nine T0 events from AGL1 were RMCE events. Based on the RMCE frequency. AGL1 produced more of the RMCE events than LBA4404 THY‐(0.12% vs. 0%). The four RMCE events identified had no accessory DNA insertions based on qPCR detection, suggesting that transient expression of *flp* resulted in SSI.

Next, we investigated the impact of maize morphogenic genes (*Bbm* and *Wus2*) on *Agrobacterium*‐mediated SSI. It is known that morphogenic genes can improve transformation frequency in maize (Lowe *et al*., 2016). We used a two T‐DNA cohabitating vector (CHV) design for testing, one with the donor DNA (donor DNA1, Table S1; spectinomycin antibiotic marker) and the second carrying the morphogenic genes (DNA2, Table S1; kanamycin antibiotic marker). These two T‐DNA plasmids reside in a single *Agrobacterium* strain. Stable integration of morphogenic genes can reduce regeneration (Lowe *et al*., 2016), therefore a stress inducible Rab17 promoter (Vilardell *et al*., 1991) driving expression of the *cre* recombinase was used to excise the *Bbm*,* Wus2* and *cre* expression cassettes flanked by *loxP* sites. Addition of morphogenic genes improved transformation frequency in both *Agrobacterium* strains, LBA4404 THY‐ and AGL1. The recovery of T0 transformants with strain LBA4404 THY‐ increased from 0.05% (binary vector) to 0.61% in the presence of the maize morphogenic genes. Similarly, AGL1 carrying the maize morphogenic genes exhibited an ~10‐fold increase in transformation frequency, from 0.27% to 2.65% (Table 1).

For experiments that included morphogenic genes, molecular characterization of T0 events was performed as described earlier with the inclusion of qPCR assays for the detection of the *Bbm* and *cre* genes. RMCE events were identified using the same criteria as described earlier, with addition of *Bbm* and *cre* genes to the list of unintended DNA insertion. A summary of molecular characterization and identification of SSI events are provided in Table S3. Five RMCE events were recovered from LBA4404 THY‐ (Table 1), our first successful recovery of SSI events utilizing this strain in these studies. Similarly, strain AGL1 carrying the maize morphogenic genes increased in RMCE frequency 10‐fold (0.12%–1.12%) as shown in Table 1.

The data presented in Table S3 indicate that RMCE events resulted from the transient expression of *flp* (#1 and #4) or the combination of *Bbm* and *flp* genes as none of the genes could be detected by qPCR (#7, #14 to #18). Taken together, these data demonstrate improved recovery of T0 transformants and RMCE events in the presence of maize morphogenic genes during *Agrobacterium*‐mediated transformation. Due to the marked improvement in recovery of both T0 transformants and RMCE events with strain AGL1, all future SSI experiments were performed using this strain carrying the maize morphogenic genes.

The strain AGL1 containing the morphogenic genes was also tested in a second maize elite inbred line, PH2RT, containing the *FRT*1*/*87 RTL for *Agrobacterium*‐mediated SSI. Three RMCE events were identified infecting over 2050 embryos suggesting very low RMCE (0.15%) frequency compared to HC69 (1.12%). The above data establishes successful *Agrobacterium*‐mediated SSI in a different maize inbred.

### Single T‐DNA donor design with morphogenic genes

The co‐transformation frequency of two T‐DNAs range anywhere >60% which can be variable in maize (Miller *et al*., 2002). Therefore, we speculated that lower co‐transformation frequency is likely to reduce the efficiency of *Agrobacterium*‐mediated SSI. To test this hypothesis, we developed single T‐DNA vectors containing the donor DNA, *flp* recombinase and excisable *Bbm*,* Wus2* and *cre* expression cassettes flanked by *loxP* sites (See Table S1, donor DNA3). The impact of the two‐TDNA vector design (donor DNA1+ DNA2) was compared to the single T‐DNA design (donor DNA3) using target line GT6. Transformation, event regeneration and molecular characterization of the T0 transformants were performed as previously described. The T0 transformation and RMCE event frequencies are summarized in Table 2. The single T‐DNA vector (donor DNA3) produced higher T0 transformation (3.9% for single T‐DNA vs. 2.9% for two T‐DNA) and RMCE (1.15% for single T‐DNA vs. 0.61% for two T‐DNA) event frequencies. An ~ twofold improvement in the frequency of RMCE event recovery was observed using the single T‐DNA vector.

**Table 2:** Comparison of the single T‐DNA and two T‐DNA vectors carrying morphogenic genes on *Agrobacterium*‐mediated SSI in target line GT6. The transformation and intended RMCE events were identified from side‐by‐side testing of the single T‐DNA (donor DNA3) and two‐T‐DNA constructs (donor DNA1+ DNA2)

Vector design	Embryos (number)	T0 transformation		RMCE	
		Events (number)	Frequency	Events (number)	Frequency
Donor DNA1+ DNA2	2269	66	2.9%	14	0.61%
Donor DNA3	2252	88	3.9%	26	1.15%

Over 100 T0 RMCE events, were subjected to SbS analysis (Zastrow‐Hayes *et al*., 2015). Only events with fully intact donor DNA insertions (flanked by intact *FRT*1*/*87 sites) and no unintended DNA sequence were considered SbS‐pass events. Using these criteria, 85% (60 of 71 T0 RMCE events) of the single T‐DNA generated events passed SbS screening, while 69% (42 of 61 T0 RMCE events) of the events from two T‐DNA passed SbS. The two T‐DNA design had significantly higher SbS fail rate (31%; *P *= 0.032). Based on the improved RMCE and SbS pass frequencies the single T‐DNA approach for *Agrobacterium*‐mediated SSI is superior.

### Testing of different *FRT* pairs

Choice of heterologous recombination sites was previously shown to improve RMCE frequency (Li *et al*., 2010; Louwerse *et al*., 2007). Compatible target sites in direct orientation with high cross‐reactivity is likely to favour excision in the presence of recombinase. If the reaction in the presence of recombinase favours excision, RMCE is likely to be lower. Therefore, heterologous *FRT* sites with low cross‐reactivity have the potential to further improve RMCE efficiency. To validate this hypothesis, three heterologous *FRT* site combinations, *FRT*1*/*6, *FRT*1*/*12 and *FRT*1*/*87, were evaluated. The *FRT* pairs described here differ in the spacer sequence (Table S4). Heterologous *FRT* pairs in direct orientation differ with respect to cross‐reactivity (self‐recombination) between themselves and can result in excision, inversion or recombination (Li *et al*., 2009, 2010). A transient screening method was developed to quantify the cross‐reactivity between the *FRT* pairs. This involved transient expression of FLP (3 or 6 days’ post‐treatment) delivered through either *Agrobacterium* or biolistic transformation (FLP plasmid DNA concentration—2.5 or 10 ng/shot), followed by qPCR determination of CT values to quantify the frequency of excision. *FRT* pairs that readily cross‐react or recombine are expected to have lower CT values, while *FRT* pairs with lower cross‐reactivity will have higher CT values.

We initially tested different DNA delivery methods to evaluate FLP‐mediated cross‐reactivity between heterologous *FRT* sites using GT6 embryos with *FRT*1*/*87 site. *Agrobacterium*‐ delivered FLP showed lower excision frequency (25%) compared to biolistically delivered FLP (50% at 2.5 ng and 100% at 10 ng) at 3‐day post‐treatment (DPT), while 100% excision was observed at day 6 with both delivery systems. This observation is consistent with the differences in the amount of DNA delivered by the two methods.

Since the biolistic delivery method allowed early detection and provided the flexibility to vary FLP concentrations to measure *FRT* cross‐reactivity, we used this method to measure excision frequency between multiple heterologous *FRT* pairs (1/6; 1/12 and 1/87) using embryos derived from independent target lines containing the *FRT*1/6, *FRT*1/12 or *FRT*1/87 sites. Two different FLP plasmid DNA concentrations (2.5 and 10 ng) and two different time points (3 and 6 days’ post‐treatment) were used for quantifying excision frequency. The data showed good correlation between the CT value, FLP DNA amount (2.5 and 10 ng), the number of days post‐treatment (3 DPT or 6 DPT) with the frequency of excision between the different *FRT* pairs tested (Table 3). Increasing amounts of FLP DNA and later time points resulted in lower CT values across the three *FRT* combinations (1/6; 1/12 and 1/87). The *FRT*1/87 pair readily excised in the presence of FLP (~100% excision irrespective of the amount of FLP DNA or time points), while lower excision rates were detected between the *FRT*1*/*6 pair (0% at 3 DPT and 8% at 6 DPT) and no excision was observed with the *FRT*1*/*12 pair in treatments with lower amounts of FLP DNA (2.5 ng, Table 3). At higher FLP concentrations, excision was detected between the *FRT*1/12 sites at 6 DPT (8%), while >30%–36% of the *FRT*1/6 pairs had recombined at the same time point (Table 3). Based on the above observations, we inferred that the *FRT*1*/*6 and *FRT*1*/*12 pairs with lower cross‐reactivity would be a better choice of *FRT* sites for improving the frequency of RMCE events.

**Table 3:** Cross‐reactivity between different heterologous *FRT* sites in the presence of FLP protein. To determine the cross‐reactivity between different *FRT* pairs (1/6, 1/12 and 1/87) embryos derived from individual target lines in the inbred HC69 containing the *FRT*1/6, *FRT*1/12 and *FRT*1/87 pairs were bombarded with two different concentrations of FLP plasmid DNA (2.5 and 10 ng) respectively. Individual embryos were collected at two different times points, 3 days post‐treatment (3 DPT) or 6 DPT and qPCR assays were performed to capture the CT (threshold cycle) values which was used to determine the frequency of excision between different *FRT* pairs

FRT site combinations and FLP plasmid DNA concentration	Mean CT value ± SD (3 DPT)*	Mean CT value ± SD (6 DPT)*	Percentage of events excised (3 DPT)†	Percentage of events excised (6 DPT)†
*FRT*1/87 (2.5)	34.48 ± 1.2	33.15 ± 1.53	97.2	100
*FRT*1/87 (10)	32.39 ± 1.05	30.73 ± 0.85	100	100
*FRT*1/6 (2.5)	40 ± 0	38.93 ± 0.35	0	8.3
*FRT*1/6 (10)	38.19 ± 1.2	37.23 ± 1.08	30.5	36.1
*FRT*1/12 (2.5)	40 ± 0	40 ± 0	0	0
*FRT*1/12 (10)	40 ± 0	39.08 ± 0.13	0	8.3

* Mean CT cycle with standard deviation values from three replicate experiments using a minimum 12 independent embryos each for the different *FRT* pairs (1/6, 1/12 and 1/87).

† Percentage of the embryos identified as excised based on CT values in the pool of 36 embryos from different *FRT* pairs (1/6, 1/12 and 1/87) bombarded with FLP plasmid DNA (2.5 and 10 ng).

### SSI across multiple RTLs

Using heterologous recombination sites, *loxP*/*loxP5*1*7*1 Louwerse and colleagues previously reported higher RMCE event recovery in Arabidopsis (Louwerse *et al*., 2007).

To evaluate the effect of heterologous *FRT* sites on RMCE frequency, we tested ≥ 6 independently generated RTL lines for three *FRT* pairs (1/6; 1/12 and 1/87) in the inbred HC69. Immature embryos were isolated and transformed with constructs containing corresponding donor DNA (donor DNAs 3, 4 or 5, Table S1) with the corresponding *FRT* pair. The three donor DNAs carried identical T‐DNAs, with the exception of the variable *FRT* pair. The transformation and RMCE frequencies are summarized in Table 4. We observed higher transformation frequencies in lines with *FRT*1*/*6 or *FRT*1*/*12 integration sites (22.5% and 19%, respectively) compared to the line with *FRT*1*/*87 integration site (4.6%). The higher transformation frequency led to a similar increase in RMCE frequency. The frequency of RMCE events was 3.5‐fold higher in lines with the *FRT*1*/*6 or *FRT*1*/*12 integration sites compared to the line with the *FRT*1*/*87 integration site. The RMCE frequencies (6.7%–6.9%) measured as proportion of the number of embryos infected are much higher than reported by Louwerse *et al*., 2007 using heterologous *loxP* sites. Noticeably >50% of the T0 events generated with *FRT*1/6 and *FRT*1/12 were RMCE events. The above data indicates the choice of *FRT* pair is important for RMCE frequency and that *FRT* pairs with lower cross‐reactivity improved RMCE event recovery in maize.

**Table 4:** Effect of different *FRT* pairs on transformation frequency and RMCE frequency. For determining the effect of *FRT* pairs on SSI, embryos derived from ≥6 target lines in the inbred HC69 containing the *FRT*1/6, *FRT*1/12 and *FRT*1/87 pairs were transformed with corresponding donor cassette to determine the T0 transformation frequency and RMCE frequency

*FRT* pair	Embryos (number)	T0 transformation frequency	RMCE	
			T0 events (number)	Frequency
1/6	462	22.5%	32	6.9%
1/12	676	19.1%	45	6.7%
1/87	3218	4.6%	39	1.2%

### Progeny analysis

From the SbS‐pass RMCE events, seven plants were selected and self‐fertilized in the greenhouse to enable segregation analysis. We evaluated T1 plants for zygosity of inserted transgene copy number (*pmi* and *DsRed*) using qPCR. Based on the segregation of the transgene copy number across the hemi, homo and null at T1 generation, all seven events showed the expected Mendelian inheritance of the transgenes in T1 generation (Table 5). In T1 generation, the plants homozygous for the transgene were selected and further selfed for seed increase. In subsequent generations none of the events showed any segregation of the transgene; they were homozygous fixed. These data confirmed the stable inheritance of the SSI locus.

**Table 5:** Observed and expected number of homozygous, hemizygous and null plants for transgene copy number in seven SbS pass events with the Chi‐square values in T1 generation

Event name	Null		Hemizygous		Homozygous		Chi‐square	*P*‐value*
	Observed	Expected	Observed	Expected	Observed	Expected		
E10347.49.2.3, EA‐3007.68.2.9	18	25	54	50	27	25	2.45	0.293758
E10347.87.3.1, EA‐3005.42.2.74	17	25	60	50	21	25	5.26	0.072078
E10427.83.3.5, EA‐3005.41.2.10	21	25	50	50	27	25	0.77	0.680451
E10602.22.5.2, EA‐2756.87.1.7	20	25	54	50	25	25	1.32	0.516851
E9846.94.1.39, EA‐3390.04.2.2	22	25	53	50	26	25	0.56	0.755784
E10347.25.1.5, EA‐2757.016.1.34	24	25	49	50	26	25	0.09	0.955997
E10427.68.4.2, E9641.99.3.1	21	25	56	50	23	25	1.52	0.467666

* Not statistically significant deviations from a 1 : 2 : 1 segregation at 5% level.

## Discussion

More than 90% of the events generated using random transformation are unsuitable for product development because of low molecular quality or the genomic insertion site. Site‐specific integration provides a solution for targeting genes to a predefined and pre‐characterized genomic location. This approach generates events with consistent gene expression (Albert *et al*., 1995; Chawla *et al*., 2006). Additionally, this technology offers the potential to create complex trait loci with multiple sites in favourable genomic locations.

An efficient method for gene insertion in maize using *Agrobacterium*‐mediated SSI is described here. The method uses a previously described promoter‐trap system flanked by heterologous *FRT* sites (Li *et al*., 2009, 2010) for successful recovery of RMCE events in maize. The use of a promoter‐trap system for SSI was first demonstrated in tobacco (Albert *et al*., 1995) and its utility was further extended to other plants (Li *et al*., 2009; Louwerse *et al*., 2007; Vergunst *et al*., 1998). The majority of work on SSI in plants has used biolistic DNA delivery (Albert *et al*., 1995; Srivastava and Ow, 2002; Srivastava *et al*., 2004). Previously reported methods of SSI are of low efficiency and therefore have not been adopted for either gene optimization or product development. The SSI frequencies reported in crops ranged from 0.3 to 0.7 events per bombarded plate in rice (Nandy and Srivastava, 2011; Srivastava *et al*., 2004), and 0.5 to 1 event per bombardment plate in soybean (Li *et al*., 2009). Additionally, the nature of complex DNA insertions, integration of interspersed copies of DNA and modifications at the integration site (Altpeter *et al*., 2005; Kohli *et al*., 2003, 2010) contributed to the lack of adoption of SSI. *Agrobacterium*‐mediated SSI has been reported in the model systems of *Arabidopsis* and tobacco, but very few site‐specific T0 RMCE plants were obtained (Nanto *et al*., 2005; Vergunst and Hooykaas, 1998; Vergunst *et al*., 1998) which also limited its adoption.

We have developed an efficient *Agrobacterium*‐mediated SSI method in maize. Lines containing heterologous *FRT* integration sites and a promoter trap with a *nptII* gene were generated. The previously described promoter‐trap system was used to select events wherein the *nptII* gene in the target line is exchanged for a different selectable marker, in this case the *pmi* gene. In the development of this method, we evaluated different *Agrobacterium* strains, different vector designs, the use of morphogenic genes and different heterologous *FRT* pairs. *Agrobacterium* strain AGL1 resulted in higher transformation frequency and generated more RMCE events than strain LBA4404 (Table 1). AGL1, a super‐virulent strain, was previously shown to enhance transient T‐DNA delivery and transformation frequency in maize (Cho *et al*., 2014; Li *et al*., 2015). Perhaps, its higher level of DNA delivery contributes to its utility in SSI. We observed that the expression of the morphogenic genes *Bbm* and *Wus2* improved frequency of transformation and recovery of RMCE events by 10‐fold, resulting in higher frequency RMCE events for both *Agrobacterium* strains AGL1 and LBA4404 THY‐. This observation is consistent with the earlier reports that co‐expression of morphogenic genes *Bbm* and *Wus2* stimulates the growth of embryogenic callus resulting in improved transformation frequencies (Lowe *et al*., 2016). Our finding complements an earlier observation where introduction of a recombinase along with the *ipt* gene was shown to stimulate selective proliferation of transgenic cells and promoted recovery of RMCE events (Ebinuma *et al*., 2015). Our data also demonstrates that RMCE events can be recovered from transient expression of *Bbm/Wus2* and *flp*, with no stable integration of these genes required.

The choice of heterologous *FRT* pair was demonstrated to be important for improving recovery of RMCE events. Umlauf and Cox (1988) showed that sequence changes in the spacer (8 bp) region of the *FRT* site can drastically reduce recombination without affecting recognition by the FLP protein. Spacer mutants with perfect self‐recombination, combined with a minimal cross‐reactivity seems to be important for enhancing RMCE (Turan *et al*., 2011). This was further validated by Lee and Saito (Lee and Saito, 1998) using spacer mutation sites *loxP5*1*7*1 which recombined with themselves but not with a wild‐type *loxP* site. Consistent with this earlier work, we observed that *FRT* cross‐reactivity was negatively correlated with RMCE frequency. Noticeably, we found that over 50% of the SSI events generated with *FRT*1/6 and *FRT*1/12 were perfect RMCE events containing the intact transgene sequence at the desired locus. This translates to a >50% increase in downstream process efficiency, since 50% of the T0 events are kept based on molecular attrition, compared to <10% using random transformation. Therefore, our preferred method uses the heterologous *FRT*1/6 pair for routine *Agrobacterium*‐mediated SSI in maize.

In conclusion, an efficient *Agrobacterium*‐mediated SSI strategy using the FLP‐*FRT* system in maize has been developed for routine transgenic event production. The application of this technology has been further extended to additional elite corn inbreds with heterologous *FRT* pairs. Our work establishes the use of FLP‐*FRT* system for generating high quality transgenic events in maize with no gene disruption at high frequencies with the potential to replace random transformation.

## Materials and methods

### DNA vector construction

DNA constructs used in the study are described in Table S1. The details of the genetic components used for vector construction is listed in Table S6. All the donor constructs were generated in the cointegrate vectors as previously described (Zhi *et al*., 2015) for maize transformation, while the morphogenic genes used in the two T‐DNA designs were built in a binary vector. The use of a promoter trap with *FRT*1 site upstream flanked by heterologous *FRT* pairs was used to aid in SSI event identification (switch in the selectable marker). Some materials reported in this paper contained reporter marker such as DsRed, AmCyan1 and selectable marker PMI owned by third parties. Similarly, some of the inbred lines and the target lines reported here are proprietary. Corteva will provide materials to academic investigators for non‐commercial research under an applicable material transfer agreement subject to proof of permission from any third‐party owners of all or parts of the material and governmental regulation considerations. Obtaining permission from third parties will be the responsibility of the requestor.

### Maize transformation and molecular event characterization

Lines with heterologous *FRT* sites were generated in the elite inbreds HC69 and PH2RT via *Agrobacterium*‐mediated transformation using a construct containing ZmProUbi‐*FRT*1‐NptII::PinII + ZmUbiPro::AmCyan1::PinII‐*FRT*87 (additional *FRT* combinations included the same donor DNA but varying FRT pairs *FRT*1*/*6 and *FRT*1*/*12, Table S1). Only single copy, backbone‐free events were selected, subjected to SbS. The hemizygous embryos derived from the RTLs were used for *Agrobacterium*‐mediated SSI. Immature embryos derived from hemizygous RTL, GT6 in the elite genotype HC69 with *FRT*1*/*87 *FRT* sites was used for optimizing *Agrobacterium*‐mediated transformation. We additionally used an RTL from PH2RT to further demonstrate SSI in a different genotype.

For comparing heterologous *FRT* sites, we used ≥6 independent RTLs in the inbred HC69. Maize immature embryo transformation was performed using split ear transformation as described in Cho *et al*., 2014 with mannose selection. Putative RMCE events were screened for DsRed expression with a fluorescence microscope (Leica) followed by selecting the transformed calli on mannose media. For gene excision an inducible Rab17 promoter driving the expression of Cre recombinase was used. This promoter can be induced by osmotic stress or through desiccation (Vilardell *et al*., 1991). The putative transgenic events were desiccated overnight on dry filter paper before moving onto maturation media to induce Cre expression and excision of the morphogenic and *cre* genes. T0 transformation frequency was determined as the number of events (plantlet with roots) produced to the total number of embryos infected. Only a single healthy‐looking event per embryo was used for determining the transformation frequency and for molecular analysis.

For molecular analysis, genomic DNA extracted from leaf punches derived from T0 events and wild‐type plants was subjected to multiplex PCR assays to detect the presence/absence of random binary vector backbone (Wu *et al*., 2014) for random T‐DNA integration, qPCR assays for detecting the presence/absence of the accessory components (*flp*,* cre* and *Bbm*) and qPCR assays to confirm excision of the target gene (*nptII*) and integration of the donor genes (*pmi* and *DsRed*). Additionally, PCR assays spanning the Ubi‐*FRT*1 (FRT 5′) and *FRT*6*/*12*/*87‐ PinII (FRT 3′) junctions were performed to confirm the presence of both FRT junctions for final identification of the RMCE events (Figure 1c). The details of the primer probe used for molecular analysis to identify the intended RMCE are described in Table S5a. The transformants identified with the intended donor DNA insertions (RMCE) were subjected to Southern‐by‐Sequencing as previously described (Zastrow‐Hayes *et al*., 2015). Events with fully intact donor DNA flanked by the heterologous *FRT* pairs absent for unintended DNA sequence were identified as SbS pass events.

### *Agrobacterium tumefaciens* culture conditions

*Agrobacterium tumefaciens* strains were grown on solidified or liquid AB Sucrose or yeast peptone medium (YP) or Luria Broth (LB) medium supplemented with appropriate antibiotics (rifampicin, 10 mg/L; spectinomycin, 100 mg/L; tetracycline 12.5 mg/L) at 28 °C. For plant transformation, *Agrobacterium* strains were streaked from glycerol stocks on AB sucrose media supplemented with appropriate antibiotics; one to three colonies were picked.

### Determination of cross‐reactivity between various *FRT* pairs

For determining the effect of transient FLP delivery on heterologous *FRT* site cross‐reactivity, immature embryos derived from target site with *FRT* pairs (1/6, 1/12 and 1/87) were used. FLP plasmid DNA was either delivered using *Agrobacterium* or biolistic transformation (2.5 and 10 ng) and immature embryos collected at 3 and 6 days’ post‐treatment (DPT), replicated thrice and used for quantification. Specific PCR primer/probe were designed (ZmUbi:FRT3′, Figure 1d and Table S5b) to quantify the excision rates between the *FRT* pairs. A minimum of 12 individual embryos were collected per treatment following *Agrobacterium* infection or biolistic delivery at different time points (3 and 6 DPT), followed by qPCR for quantifying excision frequency between the *FRT* pairs. Normalized procedures including sample collection, extraction and probe‐based PCR were used to collect the CT (threshold cycle) values in order to determine whether cross‐reactivity was occurring. Data from CT values was only used to determine whether qPCR provided a signal for amplification or lack thereof. We chose not to use a normalizer (house‐keeping gene) to maintain robustness to capture the smallest amounts of any potential cross‐reactivity.

## Conflict of Interest

AA, W.G‐K and EW are inventors on pending applications on this work and are current employees of Corteva Agriscience™ which owns the pending patent applications. ZL, ST, MA, BL, TJ and NDC are current employees of Corteva Agriscience™.

## Author contribution

A.A., E.W., W.G‐K., T.J. and N.D.C conceived the research idea and designed research; Z.L. and S.T. conducted maize transformation; M.A., B.L. and A.A. contributed new reagents and supported vector construction; E.W. and A.A, performed data analysis; J.S.M conducted progeny analysis; A.A., T.J., and N.D.C. wrote the manuscript.

## Supporting information


**Table S1** Molecular characterization of the T0 SSI events generated from two different strains of *Agrobacterium*, AGL1 and LBA4404 THY‐.
**Table S2** Molecular characterization of the T0 SSI events generated with and without morphogenic genes in the construct design for Agrobacterium‐mediated SSI.
**Table S3** The impact of expression cassette arrangement within a single T‐DNA construct on transformation and RMCE frequencies in *Agrobacterium*‐mediated SSI in the target line GT6.
**Table S4** The different FLP recognition target sites (*FRT*) and their sequences used in this study.
**Table S5** Primer pairs and probe used in this study.
**Table S6** Genetic elements used for generating expression cassettes within the T‐DNA.
