# Supplementary material for: High efficiency Agrobacterium‐mediated site‐specific gene integration in maize utilizing the FLP‐ FRT recombination system
Source: Plant Biotechnol J. 2019 Mar 28;17(8):1636–45. doi: 10.1111/pbi.13089 (PMC6662307; doi:10.1111/pbi.13089)
Supplement: Supplementary file 1 — Table S1 Molecular characterization of the T0 SSI events generated from two different strains of Agrobacterium, AGL1 and LBA4404 THY‐. Table S2 Molecular characterization of the T0 SSI events generated with and without morphogenic genes in the construct design for Agrobacterium‐mediated SSI. Table S3 The impact of expression cassette arrangement within a single T‐DNA construct on transformation and RMCE frequencies in Agrobacterium‐mediated SSI in the target line GT6. Table S4 The different FLP recognition target sites (FRT) and their sequences used in this study. Table S5 Primer pairs and probe used in this study. Table S6 Genetic elements used for generating expression cassettes within the T‐DNA. [file PBI-17-1636-s001.docx]

**Supplementary Table 1**. List of the T-DNA vectors designed for *Agrobacterium*-mediated site-specific integration.

| Cassette | Expression unit | Bacterial selection |
| --- | --- | --- |
| RTL DNA | RB- ZmUbiPro-*FRT*1-NptII::PinII + ZmUbiPro::AmCyan1::PinII-*FRT*87-LB | Spectinomycin |
| Donor DNA1 | RB-CaMV35S TERM-*FRT*1-PMI::PinII + ZmUbiPro::DsRed::PinII-*FRT*87- ZmUbiPro::Flp::PinII-LB | Spectinomycin |
| DNA2 (morphogenic genes) | RB- *lox*P -Rab17Pro::Cre::PinII+ NOS2Pro::Wus2::PinII+ ZMUbiPro::Bbm::PinII- *lox*P -LB | Kanamycin |
| Donor DNA3 | RB-ZmUbiPro::Flp::PinII: CaMV35S TERM-*FRT*1-PMI::PinII+ ZmUbiPro::DsRed::PinII-*FRT*87- *lox*P -Rab17Pro::Cre::PinII+ NOS2Pro:: Wus2::PinII+ ZMUbiPro:: Bbm::PinII- *lox*P -LB | Spectinomycin |
| Donor DNA4 | RB-ZmUbiPro::Flp::PinII: CaMV35S TERM-*FRT*1-PMI::PinII+ ZmUbiPro::DsRed::PinII-*FRT*6- *lox*P -Rab17Pro::Cre::PiniI+ NOS2Pro::Wus2::PinII+ ZMUbiPro::Bbm::PinII- *lox*P -LB | Spectinomycin |
| Donor DNA5 | RB-ZmUbiPro::Flp::PinII: CaMV35S TERM-*FRT*1-PMI::PinII+ ZmUbiPro::DsRed::PinII-*FRT*12- *lox*P -Rab17Pro::Cre::PinII+ NOS2Pro:: Wus2::PinII+ ZMUbiPro::Bbm::PinII- *lox*P -LB | Spectinomycin |

**Supplementary Table 2**. Molecular characterization of the T0 SSI events generated from two different strains of *Agrobacterium*, AGL1 and LBA4404 THY-. The first nine events were generated from AGL1 and the bottom two events from LBA4404 THY- using embryos derived from GT6 target line.

| Event | Copy number | | | | PCR (presence/absence) | | | Outcome of transformation |
| --- | --- | --- | --- | --- | --- | --- | --- | --- |
|  | *DsRed* | *pmi* | *nptII* | *flp* | *FRT*1 | *FRT*87 | Backbone |  |
| 1 | 0.61 | 0.58 | null | 1.16 | + | + | + | non-RMCE |
| 2 | 0.96 | 1.12 | null | null | + | + | - | RMCE |
| 3 | 1.07 | 1.00 | null | null | + | + | - | RMCE |
| 4 | 0.94 | 0.98 | null | null | + | + | - | RMCE |
| 5 | 0.87 | 0.84 | null | null | + | + | - | RMCE |
| 6 | null | null | null | 0.58 | - | - | + | non-RMCE |
| 7 | 0.88 | 0.53 | 0.52 | 1.76 | + | - | + | non-RMCE |
| 8 | 1.08 | 1.06 | null | null | + | - | - | non-RMCE |
| 9 | 1.99 | 2.22 | null | null | + | + | + | non-RMCE |
| 10 | null | null | null | 1.22 | - | - | + | non-RMCE |
| 11 | 1.89 | 2.12 | null | null | + | + | + | non-RMCE |

**Supplementary Table 3**. Molecular characterization of the T0 SSI events generated with and without morphogenic genes in the construct design for Agrobacterium-mediated SSI. Donor DNA1 contains no morphogenic genes, while CHV vectors (donor DNA1 + DNA2). contain the morphogenic genes (DNA2).

| Event | Construct | Copy Number | | | | PCR (presence/absence) | | | | | Outcome of the T0 event |
| --- | --- | --- | --- | --- | --- | --- | --- | --- | --- | --- | --- |
|  |  | *DsRed* | *pmi* | *nptII* | *flp* | *FRT* | | *Bbm* | *Cre* | Backbone |  |
|  |  |  |  |  |  | 1 | 87 |  |  |  |  |
| 1 | Donor DNA1 | 1.02 | 1.10 | null | null | + | + | NA | - | - | RMCE |
| 2 | Donor DNA1 | null | null | 0.87 | null | - | + | NA | - | - | non-RMCE |
| 3 | Donor DNA1 | 1.05 | 2.12 | null | null | + | + | NA | - | + | non-RMCE |
| 4 | Donor DNA1 | 1.00 | 0.96 | null | null | + | + | NA | - | - | RMCE |
| 5 | Donor DNA1 | null | null | null | 1.12 | - | - | NA | - | - | non-RMCE |
| 6 | Donor DNA1 | null | null | null | 1.00 | - | - | NA | - | - | non-RMCE |
| 7 | Donor DNA1+ DNA2 | 1.08 | 0.87 | null | null | + | + | - | - | - | RMCE |
| 8 | Donor DNA1+ DNA2 | 1.99 | 2.17 | 0.96 | null | + | + | + | + | - | non-RMCE |
| 9 | Donor DNA1+ DNA2 | 0.04 | 0.04 | null | 1.00 | + | + | - | - | + | non-RMCE |
| 10 | Donor DNA1+ DNA2 | 1.08 | 1.05 | null | null | + | + | + | + | - | non-RMCE |
| 11 | Donor DNA1+ DNA2 | 1.10 | 0.93 | null | null | + | + | + | + | - | non-RMCE |
| 12 | Donor DNA1+ DNA2 | 1.40 | 0.46 | null | 1.05 | + | + | - | - | - | non-RMCE |
| 13 | Donor DNA1+ DNA2 | 1.12 | 0.96 | null | null | + | + | + | - | - | non-RMCE |
| 14 | Donor DNA1+ DNA2 | 1.08 | 0.97 | null | null | + | + | - | - | - | RMCE |
| 15 | Donor DNA1+ DNA2 | 0.95 | 0.87 | null | null | + | + | - | - | - | RMCE |
| 16 | Donor DNA1+ DNA2 | 1.01 | 1.04 | null | null | + | + | - | - | - | RMCE |
| 17 | Donor DNA1+ DNA2 | 1.00 | 0.94 | null | null | + | + | - | - | - | RMCE |
| 18 | Donor DNA1+ DNA2 | 0.91 | 0.88 | null | null | + | + | - | - | - | RMCE |

NA- not applicable

**Supplementary Table 4**. The different FLP recognition target sites (*FRT*) and their sequences used in this study. Four different *FRT* (1/6/12/87) sites were used. The sequence of the FRT site has three 13 bp symmetry sequence in the 5’ and 3’ (black text with arrows) surrounding an 8 bp core spacer sequence (bold). The base differences in the spacer sequence in the different FRT sites compared to *FRT*1 is underlined.

| **FRT site** | **Sequence (5'-3')** |
| --- | --- |
| *FRT*1 | CGAAGTTCCTATTC CGAAGTTCCTATTC **TCTAGAAA** GTATAGGAACTTC |
| *FRT*6 | CGAAGTTCCTATTC CGAAGTTCCTATTC **TTCAAAAA** GTATAGGAACTTC |
| *FRT*12 | CGAAGTTCCTATTC CGAAGTTCCTATTC **TACATAGA** GTATAGGAACTTC |
| *FRT*87 | CGAAGTTCCTATTCC GAAGTTCCTATTC **TCCAGAAA** GTATAGGAACTTC |

**Supplementary Table 5**. Primer pairs and probe used in this study.

| Target gene | 5' Primer sequence | 3' Primer sequence | Probe sequence |
| --- | --- | --- | --- |
| *DsRed* | GGTGGAGTTCAAGTCCATCTACAT | GCTTGGCGTCCACGTAGTAGT | AGAAGCCCGTGCAGC |
| *pmi* | TGGCGAAGCGATGTTCCT | GCCACGCCTTGCAGGTAA | TTCGCTGAAACACCG |
| *nptII* | CGTTGGCTACCCGTGATATTG | GGAAGCGGTCAGCCCATT | TGAAGAGCTTGGCGGC |
| *Bbm* | CGGCGATGTCTGCTTCAA | AAGCTCTGATCCCCTCATGCT | ATCCCCCAAGATTG |
| *flp* | AGGAACTCAGAGCCCGTGC | GGTTGTCCTTGAGGAGCTGGTA | AACAGGACCGGCAAC |
| *cre* | AGTCAGGAAGAACCTCATGGACAT | CCAGGTGTGCTCGCTGAAC | TCCGCGACAGGCAA |
| *AmCyan1* | TGGCCCTGTCCAACAAGTTC | CAGCCGTCCATGTGGTAGGT | TCGGCGACGACATGA |

A) Primer probe pairs used for the molecular analysis of the intended recombination mediated exchange events.

B) Primer probe pairs used for characterizing the *FRT* junctions and quantifying cross-reactivity between the *FRT* pairs.

| Target DNA region | 5' Primer sequence | 3' Primer sequence | Probe sequence |
| --- | --- | --- | --- |
| FRT5' | TTTGCTTGGTACTGTTTCTTTTGT | TTGCACTGAGTTAATGAGTTTTTGC | TCCGAAGTTCCTATTCTC |
| FRT3' | CTGAGGATCTGGTCTTCCTAAGGA | AACCTACGAAGTCATCGGAATCA | TCCGAAGTTCCTATTCTC |
| ZmUbi:FRT3' | TTTGCTTGGTACTGTTTCTTTTGT | AACCTACGAAGTCATCGGAATCA | TGCTCACCCTGTTGTT |

**Supplementary Table 6**. Genetic elements used for generating expression cassettes within the T-DNA.

| Features | Description | References |
| --- | --- | --- |
| *ZmUbiPro* | The maize ubiquitin promoter, the 5' UTR and the first intron | Christensen et al., 1992 |
| *NptII* | Maize codon optimized Neomycin Phosphotransferase II | An unpublished Pioneer sequence |
| *PinII* | The potato proteinase inhibitor II (pinII) 3’sequence | An et al., 1989 |
| *AmCyan1* | The cyan fluorescent protein derived from *Anemonia majano*, from Clonetech | Clonetech |
| *Ds-Red* | red fluorescent protein (DsRed) derived from Discosoma, from Clontech | Matz et al., 1999 |
| *Rab17Pro* | The maize rab17 promoter and 5’ UT | Busk et al., 1997 |
| *CaMV35S TERM* | The cauliflower mosaic virus terminator | Odell et a., 1985 |
| *PMI* | The phosphomannose isomerase gene from E. col | Negrotto et al., 2000 |
| *Flp* | The flippase | An unpublished Pioneer sequence |
| *Nos2Pro* | The *Agrobacterium*-derived nopaline synthase promoter | An, 1986 |
| *Zm-Wus2* | The maize Wuschel2 (Wus2) gene | Lowe et al., 2007 |
| *Zm-Bbm* | The maize Baby boom gene (Bbm | Gordon-Kamm et al., 2005 |
| *lox*P | The recombinase target site for the CRE recombinase from *E coli* | Odell et al., 1990. |
